# Two sides of the coin: coagulation and inflammation in deep vein thrombosis – a prospective study on D-dimer and SIRI

**DOI:** 10.3389/fmed.2025.1604286

**Published:** 2025-08-11

**Authors:** Ercan Kahraman, Sirin Cetin, Meryem Cetin, Ayse Ulgen

**Affiliations:** ^1^Medical Faculty, Department of Cardiovascular Surgery, Amasya University, Amasya, Türkiye; ^2^Medical Faculty, Department of Biostatistics, Amasya University, Amasya, Türkiye; ^3^Medical Faculty, Department of Microbiology, Amasya University, Amasya, Türkiye; ^4^Department of Mathematics and Physics, School of Science and Technology, Nottingham Trent University, Nottingham, United Kingdom; ^5^Department of Biostatistics, Faculty of Medicine, Girne American University, Karmi, Cyprus

**Keywords:** C-reactive protein, D-dimer, deep venous thrombosis, Interleukin-6, systemic inflammation response index (SIRI)

## Abstract

**Background:**

Deep vein thrombosis (DVT) is a major cause of morbidity and mortality, including pulmonary embolism and post-thrombotic syndrome. This study aimed to assess the effectiveness of inflammatory indices, derived from routine laboratory parameters, in predicting DVT.

**Method:**

In this prospectively designed study, patients diagnosed with DVT through Doppler ultrasound at a tertiary healthcare center between December 2024 and February 2025, along with a control group confirmed to be DVT-free by Doppler ultrasound, were analyzed. Blood markers such as D-dimer, CRP, IL-6, and inflammatory indices (SIRI, MHR, PLR) were compared between groups. Statistical tests included chi-square, *t*-tests, logistic regression, and ROC curve analysis. Diagnostic performance was measured using odds ratios (ORs) with 95% confidence intervals (CIs).

**Result:**

Inflammatory markers (SIRI, MHR, D-dimer, CRP, IL-6) were significantly elevated in DVT cases. SIRI demonstrated high diagnostic accuracy (AUC: 0.934) with a threshold of 0.97. Combined SIRI and D-dimer analyses yielded 93% positive and 100% negative predictive accuracy.

**Conclusion:**

This study demonstrated that inflammatory markers, particularly SIRI, were elevated in patients with deep vein thrombosis (DVT) and carried high predictive value in this patient group. The combined use of SIRI and D-dimer provided high diagnostic accuracy for DVT. SIRI, a low-cost index calculable through routine blood tests, was shown to be more effective than other inflammatory markers in predicting DVT. Additionally, the combination of SIRI and D-dimer yielded high positive and negative predictive values for DVT diagnosis.

## Introduction

Deep vein thrombosis (DVT) is a major health issue, impacting 1 to 2 individuals per 1,000 annually in Europe and North America (1). Approximately 8% of adults are projected to develop DVT during their lifetime (2). Approximately 20% of patients diagnosed with DVT die within 1 year of diagnosis, either due to complications from DVT or other clinical causes (3). DVT is a complex pathological process resulting from the formation of thrombosis in the deep venous system, arising from the interplay of multiple factors. Beyond being a leading cause of preventable in-hospital mortality, it is also a significant contributor to morbidity, adversely affecting quality of life and presenting challenges in treatment. Therefore, early diagnosis, appropriate treatment duration, and effective management are crucial in reducing DVT-related mortality and morbidity (4).

The triad proposed by Virchow has clarified numerous aspects of thrombus formation to date (5). Studies have demonstrated that, in addition to the classical triggers of DVT—stasis, hypercoagulability, and endothelial dysfunction—inflammation plays a significant role in DVT pathogenesis (6–8). Inflammation acts as both a cause and a consequence of stasis, hypercoagulability, and endothelial dysfunction in DVT. This process is further amplified through the engagement of pro-inflammatory mediators and the elevated expression of thrombogenic factors. Leukocytes have been demonstrated to be essential contributors to the pathogenesis of thrombosis, both through their cellular interactions and the cytokines they release (9–11). For example, leukocytes exacerbate endothelial damage, while activated neutrophils stimulate endothelial cells, leading to the release of neutrophil extracellular traps (NETs).

In many patients diagnosed with DVT, the expected clinical presentation of DVT may not be observed (12). This discrepancy highlights the increasing need for biomarkers that can facilitate diagnosis in suspected cases and serve as a preliminary tool before imaging methods are employed. While normal results of the D-dimer test are used to rule out DVT, the search for biomarkers that can support DVT diagnosis through elevated levels is ongoing. A deeper understanding of the inflammatory mechanisms underlying DVT has brought attention to the potential of inflammatory biomarkers in this field. Recent studies have demonstrated that inflammatory biomarker levels are elevated in patients with DVT and that the Systemic Inflammatory Response Index (SIRI) may serve as a positive predictive marker for DVT diagnosis. In this context, the study conducted by Ding et al. has also established that inflammatory biomarkers can be utilized as an adjunctive method for the diagnosis of venous thromboembolism (VTE) (13).

This study aims to develop an innovative approach for assessing DVT risk utilizing routine blood test results obtained from patients presenting to the hospital. Our research evaluates the predictive capacity of the calculated SIRI for DVT presence and represents the first study in the literature to investigate the potential benefits of combining SIRI and D-dimer for DVT diagnosis. Through the integration of both biomarkers, a novel approach is presented that can significantly enhance clinical decision-making mechanisms in the DVT diagnostic process, thereby aiming to provide clinicians with a cost-effective, non-invasive, and easily applicable predictive tool.

## Materials and methods

This prospective study was conducted in accordance with STROBE guidelines. The study included patients diagnosed with DVT and individuals whose DVT diagnosis was excluded based on Doppler ultrasonography (DUS) results, all of whom presented to the cardiovascular surgery clinic of a tertiary center. The study was conducted in accordance with ethical guidelines, and approval from the relevant ethics committee was secured.

Patients exhibiting clinical signs of DVT underwent DUS (Toshiba Aplio 500, Tokyo, Japan) using a 7.5–10-MHz linear probe, performed by senior and expert radiologists. The diagnosis of DVT was established according to established guidelines (14). DUS is a routinely utilized imaging modality with high sensitivity and specificity for diagnosing DVT (15).

Participants were selected based on the following inclusion and exclusion criteria:

Inclusion Criteria:

- Individuals aged 18 years and above.
- Patients with deep vein thrombosis diagnosis confirmed by Doppler ultrasonography (DVT group).
- Individuals with deep vein thrombosis diagnosis excluded by Doppler ultrasonography (control group).

Exclusion Criteria:

- Pregnancy or postpartum period.
- Presence of malignancy.
- Inflammatory or autoimmune diseases.
- Chronic renal failure.
- Chronic liver failure.
- Active infections.
- Myocardial infarction within the last month.
- Use of immunosuppressive therapy.
- Conditions requiring intensive care unit admission due to severe systemic disorders.

Demographic data and anthropometric measurements of the study participants were collected during their initial visit. Height measurements were conducted using a fixed stadiometer while patients stood in an upright position. Body weight was measured while the patients were fasting. Waist circumference was recorded at the level of the umbilicus during the end of expiration, with patients in a standing position, using a non-elastic tape measure.

All blood tests were performed in the central laboratory of a tertiary center following standard procedures for obtaining measurement results. Complete blood counts were measured using a hematology analyzer (Sysmex XN 1000). High-density lipoprotein cholesterol (HDL-C), low-density lipoprotein cholesterol (LDL-C), and triglyceride (TG) levels were measured using the enzymatic colorimetric method (Beckman Coulter AU 5800 automatic biochemical analyzer).

High-sensitivity C-reactive protein (hs-CRP) levels were measured using the nephelometric method on a SIEMENS BN II system. D-dimer levels were measured using an immuno-turbidimetric method (Diagnostica Stago, Gennevilliers, France). Interleukin-6 (IL-6) levels were measured using an enzyme-linked immunosorbent assay (ELISA) method (Microplate reader: BIO-TEK ELX 800; Auto strip washer: BIO-TEK ELX 50). The blood test results of the patients were documented during their initial presentation. Additionally, systemic inflammatory response index (SIRI; neutrophil count × monocyte count/lymphocyte count), and monocyte-to-HDL ratio (MHR) were calculated.

### Statistical analysis

To compare between groups, categorical variables were examined using the χ^2^ test or Fisher’s exact test. The normality of data distribution was assessed using the Kolmogorov–Smirnov test. For continuous variables with a normal distribution, the independent samples *t*-test was used, whereas the Mann–Whitney *U* test was applied to variables that did not follow a normal distribution.

Multivariate logistic regression analysis was utilized to evaluate the independent associations between inflammatory markers and DVT. Odds ratios (ORs) and their corresponding 95% confidence intervals (CIs) were calculated for each variable. Receiver operating characteristic (ROC) curve analysis was performed to determine the sensitivity and specificity of inflammatory markers for predicting DVT. Statistical significance was defined as a *p*-value <0.05 for all analyses.

The optimal cutoff values for D-dimer, NLR, and SIRI were identified using ROC curve analysis. The area under the curve (AUC) and 95% CI were calculated for each marker. Logistic regression models were constructed to assess the relationship between independent variables, both individually and in combination, and venous thrombosis. These models were used to estimate sensitivity and specificity at varying cutoff points. ROC curves were generated by plotting sensitivity against 1-specificity to evaluate the diagnostic value of combining two biomarkers or a biomarker with the SIRI index.

Positive and negative predictive values were determined using logistic regression models incorporating one or more independent variables. For specific hypothesized discriminatory values of independent variables, model-derived probabilities of venous thrombosis were calculated. Furthermore, the predicted probabilities of DVT were computed for each case included in the logistic regression analysis. All statistical analyses were conducted using R software version 3.6.3 (R Foundation for Statistical Computing, Vienna, Austria) and IBM SPSS Statistics version 26.0. A two-tailed *p*-value <0.05 was considered statistically significant in all tests.

## Results

### Patient characteristics

A cumulative total of 312 individuals were evaluated for eligibility between December 2024 and February 2025, and subsequent to the implementation of the exclusion criteria, 217 individuals were incorporated into the investigation. Consequently, the final cohort consisted of a DVT group (Group 1) comprising 102 patients and a control group (Group 2) of 115 individuals in whom DVT was ruled out.

Among the participants, 100 were male and 117 were female, with a mean age of 59.95 ± 14.11 years. No significant differences were observed between the groups in terms of age, anthropometric measurements, or body mass index (BMI). The DVT group exhibited significantly higher levels of D-dimer, leukocytes, hs-CRP, and IL-6 compared to the control group (*p* < 0.001). Moreover, no differences were noted between the groups in terms of hemoglobin levels and platelet counts (*p* > 0.05) SIRI and MHR were significantly elevated in the DVT group (*p* < 0.001) (Table 1).

**Table 1:** Participants’ data.

Variable	DVT (*n* = 102)	Control (*n* = 115)	*p*-value
Gender (male/female)	61/41	39/76	***p*** **< 0.001**
Age (year)	62.14 ± 15.53	59.60 ± 11.09	0.130
BMI kg/m^2^	30.25 ± 7.33	33.41 ± 6.11	0.375
Height (cm)	164.19 ± 11.96	155.60 ± 7.57	0.135
Weight (kg)	82.11 ± 14.73	77.80 ± 15.84	0.556
Waist circumference (cm)	100.97 ± 10.23	102.40 ± 11.71	0.781
LDL mg/dl	120.63 ± 30.84	124.4 ± 48.04	**0.490**
TG mg/dl	155.53 ± 86.36	147.7 ± 74.69	0.451
HDL mg/dl	45.44 ± 13.32	50.88 ± 12.23	**0.001**
Leukocyte μ/L ×10^3^	8.85 ± 2.96	7.24 ± 2.21	***p* < 0.001**
Neutrophil μ/L ×10^3^	5.99 ± 2.67	4.47 ± 1.95	***p* < 0.001**
Lymphocyte μ/L ×10^3^	1.91 ± 0.68	2.12 ± 0.72	**0.037**
Monocyte u/L ×10^3^	0.69 ± 0.27	0.45 ± 0.15	***p* < 0.001**
Hemoglobin g/dL	13.19 ± 1.94	13.06 ± 1.86	0.064
Platelet μ/L ×10^3^	237.5 ± 87.82	259.13 ± 74.05	0.060
SIRI	2.77 ± 3.03	1.20 ± 1.27	***p* < 0.001**
MHR	0.016 ± 0.008	0.009 ± 0.004	***p* < 0.001**
D Dimer μg/dl	4.79 ± 4.16	0.33 ± 0.21	***p* < 0.001**
hs-CRP mg/l	41.39 ± 11.04	2.85 ± 5.29	***p* < 0.001**
IL-6 pg./mL	39.90 ± 31.73	6.20 ± 3.25	***p* < 0.001**

Data are presented as the mean ± SD or as numbers and percentages.

BMI, body mass index; DVT, deep venous thrombosis; HDL, high-density lipoprotein; hs-CRP, high-sensitivity C-reactive protein; IL-6, interleukin-6; LDL, low-density lipoproteins; MHR, monocyte to high-density lipoprotein ratio; SIRI, systemic inflammation response index; TG, triglyceride.

### SIRI and DVT risk

To evaluate the predictive power of inflammatory markers in patients with DVT, ROC analysis was performed. This analysis determined the sensitivity and specificity of laboratory parameters and calculated AUC and optimal cutoff values for diagnosing DVT.

**Figure 1:**
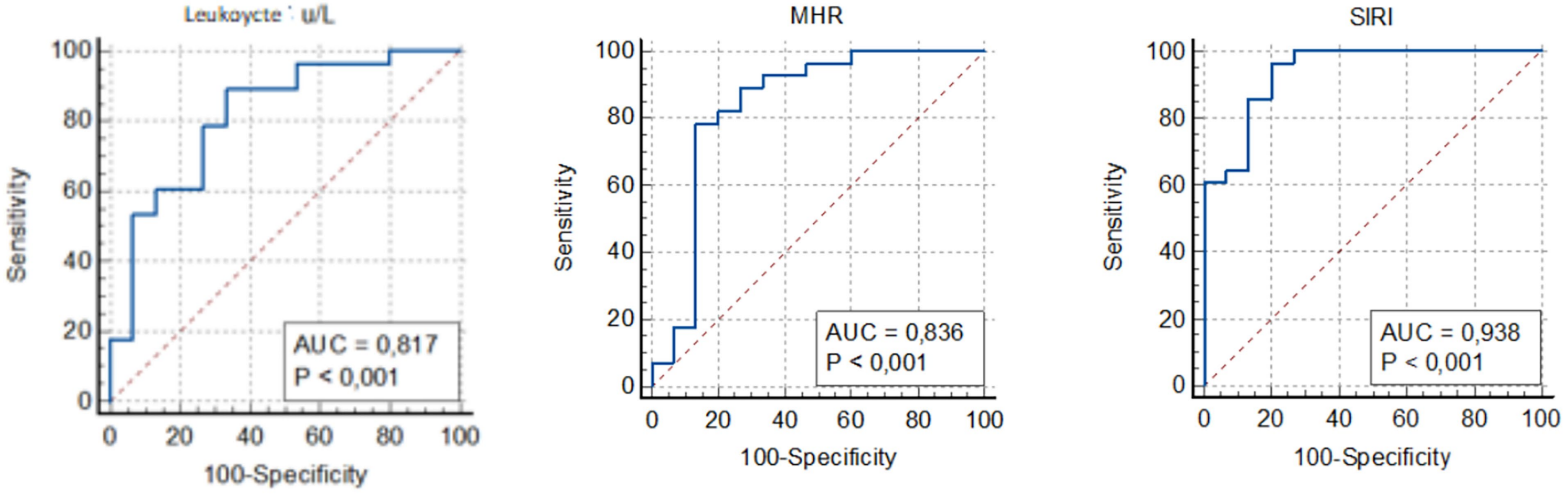
Receiver operating curve for determining the optimal cut-off values for leukocyte, MHR and SIRI. MHR, monocyte to HDL-cholesterol ratio; SIRI, systemic inflammation response index.

The AUC for leukocyte count was calculated as 0.817 (95% CI, 0.707–0.862), for D-dimer as 0.981 (95% CI, 0.933–0.998), and for MHR as 0.836 (95% CI, 0.688–0.847). The AUC for SIRI was found to be 0.938 (95% CI, 0.829–0.947) (Figure 1). Based on the ROC analysis, the optimal cutoff value for SIRI was determined to be 0.97 (sensitivity 90%, specificity 81%), whereas the optimal cutoff for D-dimer was 0.86 (sensitivity 90%, specificity 92%). These findings underscore the high accuracy of SIRI in predicting DVT.

### Combined analysis of SIRI and D-dimer

Univariate and multivariate regression analyses were conducted to examine the relationship between inflammatory markers and DVT. In the univariate logistic regression analysis, the risk of DVT associated with each inflammatory marker was evaluated, and the significant variables were included in the multivariate logistic regression analysis (Table 2). The multivariate logistic regression analysis revealed that for patients with D-dimer values above 0.86, each unit increase in D-dimer was associated with approximately a 5-fold increased risk of developing DVT (OR: 5.06, 95% CI: 1.79–14.33). Similarly, for patients with SIRI values above 0.97, each unit increase was found to be approximately 4 times more likely to be associated with an increased DVT risk (OR: 4.23, 95% CI: 1.33–13.86).

**Table 2:** Multivariate logistic regression model for the association between DVT and inflammatory markers.

Variable	Odds ratio	(95% CI)	*p*-value
SIRI	4.23	1.33–13.86	**0.005**
D Dimer ᶙg/dl	5.06	1.79–14.33	**0.001**
MHR	1.40	1.45–3.91	**0.009**
hs-CRP mg/l	1.29	1.08–1.55	**0.005**
IL-6 pg./mL	1.38	1.08–1.76	**0.008**

hs-CRP, high-sensitivity C-reactive protein; IL-6, Interleukin-6; MHR, monocyte to high-density lipoprotein ratio; SIRI, systemic inflammation response index.

The results of the combined multivariate logistic regression analysis indicated that the inclusion of SIRI values alongside D-dimer improved the predictive value of both variables. The AUC for the combination of D-dimer and SIRI was 99% (*p* < 0.0001). The multivariate logistic regression analysis demonstrated that the combination of D-dimer and SIRI (below 0.86 μg/mL and above 0.97) achieved 100% sensitivity and 93% specificity for diagnosing DVT, with a 100% negative predictive value and a 96.38% positive predictive value (Table 3).

**Table 3:** ROC area, *p*-value, sensitivity and specificity, PPV and NPV derived from a multivariate logistic regression model with single or two combined biomarkers as independent variables.

Combined multivariate logistic regression						
Variables	AUC	Sensitivity (%)	Specificity (%)	NPV	PPV	*p*-value
D-DIMER + SIRI (≤0.86 μg/ml + ≥0.97)	0.99	100	93	100	96,38	***p* < 0.001**
D-DIMER (≤0.86 μg/ml)	0.98	96,43	55,78	96,68	53,88	***p* < 0.001**
SIRI (≥0.97)	0.93	86,9	89	78,45	93,64	***p* < 0.001**

NPV, negative predictive value; PPV, positive predictive value; SIRI, systemic inflammatory response index.

The model attempts to distinguish DVT cases from non-DVT cases in a sample of cases.

### Subgroup analyses

**Figure 2:**
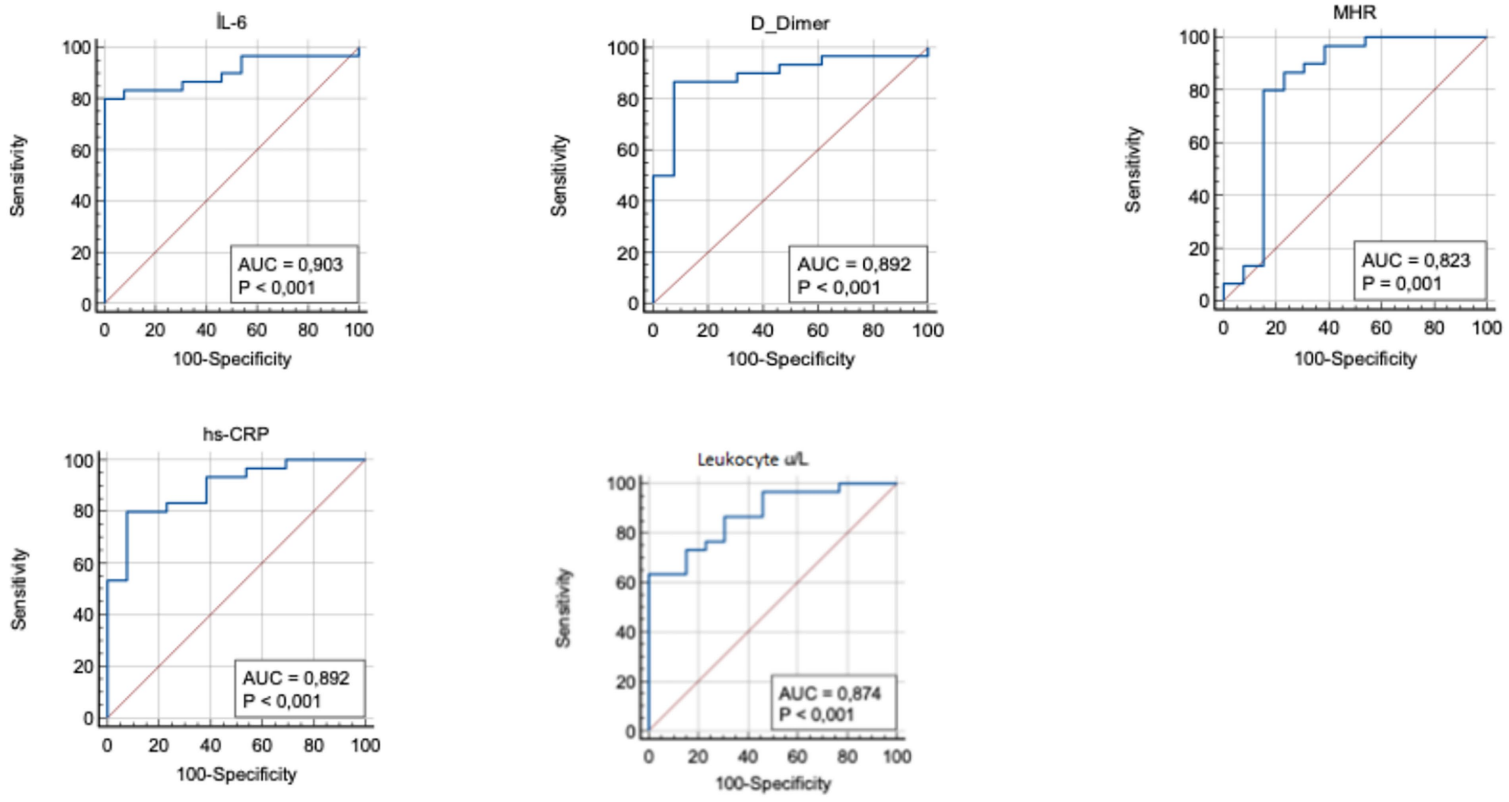
Receiver operating curve for determining the optimum cut-off values for IL-6, D-dimer, MHR, hs-CRP and leukocyte. hs-CRP, high-sensitivity C-reactive protein; IL-6, Interleukin-6; MHR, monocyte to HDL-cholesterol ratio.

To further investigate the effectiveness of SIRI in DVT patients, the patients were divided into two groups based on the SIRI cutoff value (0.97), and subgroup analyses were performed (Table 4). In patients with SIRI values above 0.97, CRP, IL-6, and D-dimer levels were significantly higher (*p* < 0.001). This finding suggests that elevated SIRI in DVT patients is indicative of a severe inflammatory response. ROC analysis was repeated for the subgroups, and the AUC and optimal cutoff values for determining the effectiveness of the SIRI index in predicting DVT are presented in Table 5. The AUC for D-dimer was found to be 0.89 (95% CI, 0.760–0.966), for IL-6 0.90 (95% CI, 0.773–0.972), for MHR 0.82 (95% CI, 0.677–0.922), for hs-CRP 0.89 (95% CI, 0.760–0.966), and for leukocyte count 0.87 (95% CI, 0.738–0.956) (Figure 2).

**Table 4:** Participants’ data generated according to SIRI cut-off value.

Variable	SIRI < 0.97 (*n* = 91)	SIRI > 0.97 (*n* = 126)	*p*-value
Gender (male/female)	64/27	53/73	0.106
Age (year)	61.09 ± 10.81	62.16 ± 14.07	0.546
Height (cm)	154.25 ± 9.70	164.07 ± 11.61	0.119
Weight (cm)	80.75 ± 12.84	81.54 ± 15.18	0.922
BMI kg/m^2^	35.76 ± 4.45	30.04 ± 7.24	0.137
LDL mg/dl	126.87 ± 43.27	119.73 ± 41.48	0.188
TG mg/dl	153.37 ± 74.39	149.29 ± 83.77	0.679
HDL mg/dl	50.58 ± 12.44	47.14 ± 13.05	**0.036**
Leukocyte μ/L ×10^3^	6.32 ± 1.53	9.11 ± 2.81	***p* < 0.001**
Neutrophil μ/L ×10^3^	4.75 ± 2.24	5.51 ± 2.53	**0.024**
Lymphocyte μ/L ×10^3^	2.30 ± 0.66	1.82 ± 0.67	***p* < 0.001**
Monocyte μ/L ×10^3^	0.61 ± 0.27	0.61 ± 0.27	**0.001**
Hemoglobin g/dL	13.22 ± 1.90	13.05 ± 1.89	0.517
Platelet μ/L ×10^3^	247.95 ± 65.68	249.97 ± 90.90	0.856
MHR	0.010 ± 0.006	0.013 ± 0.008	***p* < 0.001**
D Dimer μg/dl	0.59 ± 1.32	2.42 ± 3.67	***p* < 0.001**
hs-CRP mg/l	6.65 ± 17.40	25.88 ± 41.57	***p* < 0.001**
IL-6 pg./mL	6.01 ± 3.01	37.74 ± 31.72	**0.001**

BMI, body mass index; BUN, blood urea nitrogen; GFR, glomerular filtration rate; HDL, high-density lipoprotein; hs-CRP, high-sensitivity C-reactive protein; IL-6, Interleukin-6; LDL, low-density lipoproteins, MHR, monocyte to high-density lipoprotein ratio; PLR, platelet to lymphocyte ratio; SIRI, systemic inflammation response index; TG, triglyceride.

**Table 5:** The predictive ability of, D-dimer, IL-6, MHR, hs-CRP and leukocyte, SIRI elevation.

Variables	AUC	95% CI	Sensitivity (%)	Specificity (%)	Youden index	Cut-off value	*p*-value
D Dimer μg/dl	0.89	0.760–0.966	86.67	92.31	0.78	0.86	*p* < 0.0001
IL-6 pg./mL	0.90	0.773–0.972	80.00	98.00	0.80	10.45	*p* < 0.0001
MHR	0.82	0.677–0.922	80.00	84.62	0.64	0.001	0.0005
hs-CRP mg/l	0.89	0.760–0.966	80.00	92.31	0.72	6.92	*p* < 0.0001
Leukocyte	0.87	0.738–0.956	63.33	98.00	0.63	7.73	*p* < 0.0001

hs-CRP, high-sensitivity C-reactive protein; IL-6, Interleukin-6; MHR, monocyte to high-density lipoprotein ratio.

The results of the multivariate logistic regression analysis for subgroups revealed that in individuals with high SIRI values, when D-dimer levels were above 0.86, each unit increase in D-dimer was associated with approximately a 3-fold increase in the risk of developing DVT (OR: 3.03, 95% CI: 1.25–7.30) (Table 6). These findings suggest that SIRI, like D-dimer, may play a significant role in detecting DVT, and when evaluated together, they can predict the disease with higher sensitivity.

**Table 6:** Multivariate logistic regression model for the association between SIRI levels and inflammatory markers.

SIRI Levels			
Variable	Odds ratio	(95% CI)	*p*-value
D Dimer μg/dl	3.033	1.259–7.304	**0.013**
hs-CRP mg/l	1.182	1.046–1.336	**0.007**
IL-6 pg./mL	1.346	1.069–1.694	**0.011**

hs-CRP, high-sensitivity C-reactive protein; IL-6, Interleukin-6.

## Discussion

This study demonstrates that inflammatory biomarker levels are elevated in DVT patients, and that the SIRI may serve as a positive predictive value for DVT. Additionally, it was found that when SIRI is assessed in conjunction with D-dimer, both positive and negative predictive values for DVT diagnosis are significantly higher. This study is the first to emphasize the potential advantages of combining SIRI and D-dimer values for the diagnosis of DVT in the literature.

It is well-known that certain inflammatory markers are elevated in DVT patients (16, 17). The identification of the relationship between DVT and inflammation has led to an increase in research in this area (10). Roumen-Klappe et al. (18) demonstrated elevated levels of both CRP and IL-6 in DVT patients in their studies. There is a strong correlation between plasma levels of CRP and IL-6, as CRP gene transcription is largely dependent on IL-6 levels (19). Therefore, the inflammatory process in DVT patients leads to the elevation of both CRP and IL-6. Consistent with previous studies, the present study also found that both IL-6 and CRP levels were elevated in DVT patients.

In addition to inflammatory markers, it has been shown that inflammatory indices such as SIRI and MHR, which are calculated using simple laboratory tests, are elevated in patients with DVT (20, 21). SIRI has been identified as a strong and independent biomarker for predicting disease severity and prognosis in various clinical conditions, including cardiovascular, neurological, and oncological disorders. Elevated SIRI levels have been associated with poorer clinical outcomes and a more aggressive disease course across different diseases. Therefore, SIRI is increasingly being recognized as a relevant parameter in clinical decision-making processes. Ling et al. (20) determined the optimal cut-off value for SIRI as 0.621 in DVT patients with tibial fractures, with a sensitivity of 0.853 and a specificity of 0.379. Similarly, Liu et al. (21), in a large-scale study involving hospitalized patients, identified the optimal SIRI cut-off value as 0.65 in DVT patients, with a sensitivity of 0.72 and a specificity of 0.59. In our study, consistent with these findings, we determined the cut-off value for SIRI as 0.97, with a sensitivity of 0.86 and a specificity of 0.89. Our results indicated a cut-off value close to those previously reported but demonstrated higher sensitivity and specificity. The study by Ling et al. (20) involved patients with tibial fractures caused by trauma, a factor known to influence the inflammatory response (22), On the other hand, the population in Liu et al.’s study included hospitalized patients with other underlying diseases, which may have impacted the inflammatory parameters. These differences could explain the variations in sensitivity and specificity observed in our study. Unlike the aforementioned studies, the present study was conducted among outpatients with no history of trauma.

Logistic regression analysis to evaluate the risk posed by elevated SIRI levels in DVT patients revealed that when SIRI exceeded 0.97, each unit increase in SIRI was associated with approximately a fourfold higher risk of DVT. This observed risk increase aligns with previous studies that reported a 4.16 and 3.25 fold increase in risk, respectively (21, 23). Although elevated CRP and IL-6 levels were also detected in the DVT patient group, these biomarkers were associated with a comparatively lower risk increase of 1.29 and 1.38, respectively. While CRP and IL-6 are recognized as significant inflammatory markers, the higher risk associated with increased SIRI levels suggests that SIRI holds a distinct and notable significance among inflammatory markers, particularly in DVT patients.

The same analysis revealed that each unit increase in D-dimer above the established threshold value of 0.86 was associated with a fivefold increased risk of DVT. D-dimer is a crucial biomarker commonly used for its high negative predictive value and sensitivity to rule out the presence of DVT (24). However, elevated D-dimer levels can also occur in various conditions and diseases, such as pregnancy, malignancy, and aging, which reduces its specificity in diagnosing DVT (4). Therefore, D-dimer has traditionally been used in conjunction with ultrasonography for diagnosing DVT (25). It has also been suggested that combining D-dimer with various other biomarkers could improve its diagnostic utility (24). In line with this, a combined multivariate logistic regression analysis was conducted to better understand the predictive value of SIRI and D-dimer. The results showed that the combined use of SIRI and D-dimer could predict DVT with a positive predictive value of 96.38% and a negative predictive value of 100%. The joint evaluation of these two markers enhances diagnostic accuracy by compensating for the reduced specificity of D-dimer in DVT patients. Additionally, this approach may serve as a valuable tool in supporting DVT diagnosis, especially in suspected cases without typical DVT symptoms, allowing for assessment before Doppler ultrasonography.

To further investigate the relationship between SIRI and the inflammatory mechanisms in DVT patients, subgroup analyses were conducted based on the threshold value of 0.97 identified for SIRI. In these analyses, D-dimer and other inflammatory markers were found to be significantly higher in the group with elevated SIRI levels. Furthermore, logistic regression analysis revealed that when elevated SIRI was considered a risk factor, each unit increase in D-dimer above 0.86 was associated with a threefold higher risk of elevated SIRI. This finding highlights the strong relationship between elevated SIRI levels and increased D-dimer.

The existing literature and the findings of this study demonstrate that inflammation is associated with various clinical conditions and that this inflammatory process can be effectively monitored using the SIRI. However, many clinical conditions that warrant evaluation from this perspective have not yet been adequately investigated. For instance, although Antiphospholipid Syndrome (APS) is known to be associated with an inflammatory process, comprehensive studies examining the relationship between SIRI and APS remain limited (26). Similarly, further research is needed to explore numerous other clinical conditions from this point of view. In this context, a systematic investigation of such clinical conditions in relation to SIRI may provide valuable insights to the current body of literature.

This study has several strengths. First, it was conducted among outpatients visiting the hospital, excluding individuals with trauma or comorbidities requiring hospitalization that could alter inflammatory markers. This approach allows for a clearer depiction of the interaction between DVT and inflammation. Second, as both the DVT and control groups were assessed using Doppler ultrasonography, the findings related to the presence or absence of thrombotic events can be considered reliable. Third, various statistical analyses (e.g., Combined Multivariate Logistic Regression, subgroup analyses based on SIRI values) were performed, yielding consistent and parallel results, thus demonstrating the robustness and reliability of the findings. This study has certain limitations that should be taken into consideration. First, as it was conducted in a single center, the generalizability of the findings to broader demographic populations may be limited. Second, the cross-sectional design confines the assessment of laboratory parameters to the time of DVT diagnosis, thereby restricting the analysis of temporal changes in inflammatory markers. Third, the absence of a separate validation cohort represents a limitation, and the findings require confirmation in independent patient populations.

## Conclusion

While the inflammatory mechanisms underlying DVT remain incompletely understood, this study highlights a significant association between DVT and inflammation. The findings suggest that inflammatory indices may possess predictive potential strong enough to aid in identifying DVT. Specifically, the results emphasize that combining SIRI, a marker with a positive predictive value, with D-dimer, known for its negative predictive value, achieves high specificity and sensitivity in detecting DVT. This combined approach could enhance diagnostic accuracy and provide a more reliable framework for assessing patients with deep vein thrombosis.

## Data Availability

The datasets for this study are not provided as the participants of this study did not give written consent for public sharing of their data. Requests to access the datasets should be directed to Ercan Kahraman, kahraman@amasya.edu.tr.
